# A Comparison of Patients’ and Physicians’ Knowledge and Expectations Regarding Precision Oncology Tests

**DOI:** 10.3390/curroncol29120780

**Published:** 2022-12-16

**Authors:** Navdeep Dehar, Tasnima Abedin, Patricia Tang, Gwyn Bebb, Winson Y. Cheung

**Affiliations:** 1 Department of Medical Oncology, Queen’s University, Kingston, ON K7L 5P9, Canada; 2 Clinical Research Unit, Tom Baker Cancer Centre, Calgary, AB T2N 4N2, Canada; 3 Department of Medical Oncology, Tom Baker Cancer Centre, Calgary, AB T2N 4N2, Canada

**Keywords:** precision oncology, tumour marker, communication

## Abstract

(1) Background: As genomic testing is becoming a part of the mainstream oncology practice, it is vital to ensure that our patients fully understand the implications of these tests. This study aimed to compare the attitudes and expectations of cancer patients with those of their physicians regarding the role of biomarker testing in clinical decision making. (2) Methods: Two separate, complimentary, self-administered questionnaires for patients with cancer and their physicians, respectively, were collected in Calgary, Alberta, Canada. Out of 117, 113 completed patient surveys were included in the statistical analysis, constituting a 96.4% response rate. These surveys were subsequently matched with those of their corresponding oncologists to determine the concordance rates. (3) Results: Overall, patients demonstrated a good understanding of general cancer biology (80.0%) and diagnostic processes (90.0%) associated with precision oncology. Most patients wanted their tumours to be tested to guide treatment, and the oncologists broadly shared these views (concordance 65.1%). However, there were discrepancies between the knowledge and expectations regarding the applications of test results on actual diagnosis and prognosis between patients and their oncologists (concordance 26.1% and 36.0%, respectively). While only 28.0% of patients thought they had enough knowledge to make informed decisions, the majority (68.0%) said they needed more information. (4) Conclusion: Our study shows that patients and cancer physicians do not always agree with the roles and applications of genomic tests, which could lead to misplaced expectations and poor health outcomes. More research is needed to devise strategies to improve education and communication to align these expectations and improve the quality of clinical decision making.

## 1. Introduction

Oncology has witnessed unprecedented growth in the understanding of aberrant molecular pathways implicated in tumourgenesis [1]. Advances in high-throughput genome sequencing technology, including next-generation sequencing (NGS), have enabled scientists to identify molecular characteristics associated with disease surveillance, treatment, and clinical outcomes [1,2]. Genomic testing in cancer detects significant somatic mutations to predict disease trajectory more accurately and to improve clinical outcomes through the development of guided personalized therapies [3,4]. Greater use of such testing to drive treatment decisions regarding solid tumours has enabled the shift in cancer diagnosis and therapy from empirical to precision oncology, intending to deliver the right cancer treatment to the right patient at the right dose and at the right time [3,4].

Precision oncology testing is now a routine part of the initial assessment of many tumour types. For instance, in lung cancer, detecting EGFR mutations and ALK or ROS1 fusions helps to determine if a tyrosine kinase inhibitor (TKI) should be used instead of cytotoxic chemotherapy [5]. Additionally, PD-L1 expression (tumour proportion score of ≥50%) serves as a biomarker of the response to immunotherapy [6,7,8] in the lung, breast and many other cancer types. Similarly, among patients with metastatic colorectal cancer (mCRC), a RAS mutation is used as a predictive biomarker to select patients who will not benefit from epidermal growth factor receptor (EGFR) antibody therapy [9,10,11]. Such treatments driven by genomic testing have produced a marked increases in survival by altering the treatment decisions in approximately 50% of patients [6,7,8]. Furthermore, studies have shown that the successful use of these approaches can be safe and is independently associated with improvement across many outcome variables [11,12,13,14]. 

Despite the potential benefits of genomic testing, there is a growing clinical challenge to provide customized patient care based on complex information. Communication with patients has always been taxing in oncology, and applying genomic information in clinical decision making adds to this complexity. Therefore, it is essential to ensure that patients fully understand the role of the testing and how it may affect management decisions. Prior research has reported an insufficient understanding of genomic testing and its applications among those who received such testing for breast cancer susceptibility [15,16,17,18,19]. There is also evidence that patients with cancer have an inadequate understanding of somatic and germline mutations in genomic testing, as well as apprehensiveness towards receiving test results [15,20]. However, studies of patient knowledge and expectations of genomic testing are limited, so it remains unclear how the perspectives of patients and physicians differ regarding the use of this new technology.

Most physicians endeavour to provide their patients with enough information about genomic testing and targeted treatments to make informed decisions. However, they do not reliably know how well their patients have assimilated this information. Previous studies of patient and physician expectations of cancer care have revealed significant discrepancies [21]. For example, a survey comparing expectations for cancer survivorship care reported meagre agreement rates [21]. Comparable results were also reported in a similar study published a year later [22]. A multinational survey of oncologists and patients with cancer demonstrated regional variations in the knowledge and clinical applications of personalized medicine [23]. Such discrepancies in patient and physician knowledge and expectations can often result in suboptimal cancer management and follow-up care [20]. Increasing patient–physician communications in areas showing disagreement may lead to better concordance and a higher likelihood of receiving proper care [24,25].

With the increasing number and frequency of genomic tests currently being offered to cancer patients, it is essential to ensure that they understand the implications of this new technology. Furthermore, knowledge about the areas of agreement and disagreement between patients and providers can assist in developing strategies for improving education and counselling regarding genomic testing [26,27,28,29,30]. This paper presents the results of a pilot study comparing the self-reported knowledge and expectations regarding genomic testing and identifies differences in their views.

## 2. Materials and Methods

The study was conducted at the Tom Baker Cancer Center in Alberta, Canada. Two separate but complementary self-administered questionnaires were developed and distributed to 117 patients and 15 medical oncologists over 12 weeks between July and September 2019. The study was designed as a pilot project, with a small number of patients. The Research Ethics Board of the institution approved the study protocol and documents.

The participating oncologists were asked to complete the physician survey after informed consent. Physicians were asked to identify eligible patients based on the study inclusion criteria, which consisted of adult patients who had a primary diagnosis of cancer and had received a diagnostic test to find a targetable mutation. The physicians approached prospective patients during clinic hours, who then completed the survey questionnaire after providing informed consent. The surveys could be completed in the clinic or taken home and returned via email or Canada post.

### 2.1. Patient Questionnaire

The patient survey was designed to collect information about their demographics, cancer diagnosis, prior cancer experience, and research interests. It comprised three sections: (a) the patient’s basic knowledge and awareness of cancer and diagnostic tests, (b) the patient’s attitude toward precision oncology and testing, and (c) the patient’s acceptance of integrated testing as a part of disease management, including diagnosis and treatment. The responses to these questions were evaluated in either a binary fashion (“Yes” or “No”) or using a 5-point Likert scale (“Strongly Disagree”, “Disagree”, “Neither Agree nor Disagree”, “Agree”, “Strongly Agree”). The responses were designed to reflect the patient’s understanding, opinion, and acceptance level for genomic testing to help guide their cancer management. For details, please refer to the patient survey in Supplementary File S1.

### 2.2. Physician Questionnaire

The physicians were subsequently asked in their survey version to indicate their attitude towards genomic testing, its applications, and how much they used it in their clinical practice. They also identified factors influencing their willingness or disapproval to pursue such testing. Physicians then indicated their perception of the patient’s desire to pursue genomic testing and their concerns about a major or minor procedure undertaken during the testing process. The physician questionnaire consisted of 6 questions, which evaluated their responses on Likert scales similar to those used for the patient survey. For details, please refer to the physician survey in Supplementary File S2.

### 2.3. Statistical Analysis

Baseline demographics and characteristics for the patient and physician cohorts were summarized with descriptive statistics. Surveys with at least 50% completed responses were included in the data analysis. Each patient’s answers were matched with their corresponding oncologist to form patient–oncologist dyads, and concordance rates were determined. Since the survey responses of the oncologists did not vary from patient to patient, we matched multiple patients to their corresponding oncologists. For patient–oncologist dyads, concordance was defined as a complete agreement in response categories. IBM SPSS 21 statistical software was used for analysis.

In contrast, discordance was signified by any disagreement in response categories between the respondents of a matched survey pair. The patient’s literacy level (at or above high school level) and income (</≥CAD 50,000) were also compared with their knowledge of genomic testing using the Pearson chi-squared test. A value of <0.05 was considered significant. For the knowledge responses, we randomly selected two questions (Q15 a and b) as the best representative of patient and physician knowledge comparison from the survey: Q15 (a) “Genomics testing based on today’s technology would significantly improve the DIAGNOSIS of my cancer.” Agree or disagree (reduced category for statistical analysis); (b) “Genomics testing based on today’s technology would significantly improve the TREATMENT of my cancer.” Agree or disagree. (Refer to the patient survey in Supplementary File S1).

## 3. Results

(a) Characteristics of patients and physicians:

Out of 117 surveys distributed among cancer patients, 113 completed surveys were included in the analysis, constituting a 96.4% response rate (Table 1). A total of 15 physicians were approached; 12 were able to participate in the study and were included in the statistical analysis. Among the physicians who participated, 11 physicians were trained medical oncologists, and one was a general practitioner in oncology (GPO). The majority of the physicians were treating at least two tumour sites.

In the patient cohort, the study population consisted of 53.9% (*n* = 61) males and 46.0% (*n* = 52) females. The median age was 63 yr (range 33–93 yr), and 54.9% (*n* = 62) of patients had a family history of cancer. We chose different tumour sites to include a diverse patient population. Tumour site distribution among the patients was as follows: 44% (*n* = 48) breast, 26.5% (*n* = 30) colon, 4.4% (*n* = 5) pancreas, 3.5% (*n* = 4) renal, 9.7% (*n* = 11) melanoma, 8.8% (*n* = 10) lung, and 4.4% (*n* = 5) prostate. Nearly 37% (*n* = 42) of patients had a university-level education, while 36.3% (*n* = 41) had college-level degrees or diplomas. While 34.9% (*n* = 39) of patients had a family member working in healthcare, only 18.6% (*n* = 21) had healthcare or cancer-related experience. Approximately 70.7% (*n* = 80) reported an income of >CAD 50,000. Other demographic characteristics are illustrated in Table 1.

**Figure 1:**
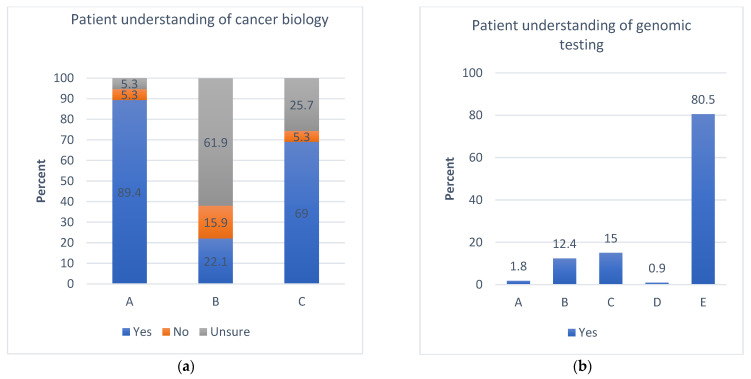
Patient survey responses regarding general knowledge of cancer and its testing. (**a**) A. Cancer represents uncontrolled cell growth; B. Genomics testing can be used to help predict how long a patient might live with cancer; C. Genomics testing can be used to help predict a patient’s response to a specific cancer drug. (**b**) Genomics testing (for acquired and inherited mutations) can potentially be used for which of the following: A. To confirm the type of cancer diagnosis, B. To predict the risk of cancer for an individual and their children or relatives, C. To select treatment and monitor a patient’s response to therapy, D. To predict disease outcome, E. All of the above.

(b) Patient understanding of cancer biology and awareness of genomic testing:

Patients were asked questions to examine their understanding of cancer biology, as well as factors contributing to tumour formation, including hereditary, lifestyle, and environments and how genomic testing can help identify tumour characteristics and guide therapy. Overall, the majority (89.4%, *n* = 101) of patients demonstrated a good understanding of what cancer means and the factors that could contribute to its causation. Out of these, more than 60.0% (*n* = 70) of patients did not know that genomic changes could lead to tumourgenesis, and only a few (25.7%, *n* = 29) knew about the role of genomic testing in prognostication. However, over 80.5% (*n* = 91) of patients understood the uses of genomic testing, including its role in affecting treatment choices and predicting patients’ responses to various treatments (Figure 1a,b). Evaluation of the relationship between the patient literacy and their knowledge of genomic testing showed no significant association, with *p* values of 0.82 and 0.57, respectively, for Q15 (a) and (b) (please refer to the patient survey and Supplementary Table S1A). Similar results were seen with patients’ income levels and understanding of genomic testing (*p* = 0.15 and *p* = 0.09, respectively (please refer to the patient survey and Supplementary Table S1B).

**Figure 2:**
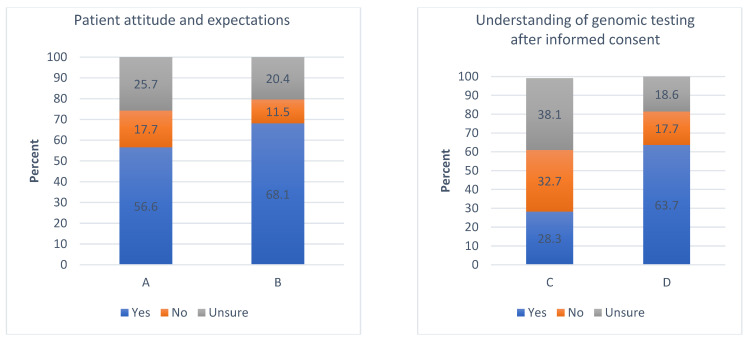
Patient responses to questions (in percentages) for attitude and expectations towards genomic testing and research. (**A**). I would only want to receive genomics test results that would influence my current or future cancer treatment decisions. (**B**). For example, I would want to receive genomics test results about my family’s inherited risk of developing cancer. (**C**). Do you think you have sufficient knowledge regarding the potential benefits and risks of genomics testing in cancer care to make an informed decision to pursue testing? (**D**). Would you need more formal counselling before pursuing genomic testing in your cancer care?

(c) Attitude and expectations of patients towards genomic testing and research:

Patients were also asked questions to understand their comfort level towards receiving unexpected results from genomic testing, which could have health and reproductive implications and impact their decision to pursue the testing [31]. As depicted in Figure 2A,B, 56.6% (*n* = 64) of patients wanted to undergo testing only for their personal use, and a large number (68.1%, *n* = 77) were also willing to know about their family’s risk of carrying a mutation that could predispose them to a similar disease. Furthermore, patients were asked about their willingness to biobank their tumour samples for future research, and more than 60.0% (*n* = 76) consented. While only 28.3% (*n* = 32) felt that they had enough information to make an informed decision after the informed consent (Figure 2C), more than 60% (*n* = 72) expressed their willingness to have more formal counselling before pursuing genomic testing for their cancer management (Figure 2D).

(d) Attitudes and expectations of patients vs physicians regarding genomic testing:

We asked patients about their willingness to undergo a major or minor procedure for genomic testing, and this was compared to the physician’s views. Less than 70.0% (*n* = 78) of patients were willing to undergo minor procedures, which was over-estimated by their physicians in nearly 82.0% (*n* = 9) of cases. However, many patients felt that their tumours should be tested to guide treatment (70.0%, *n* = 78), and these views were broadly shared by their oncologists (concordance 65.1%, Table 2).

**Figure 3:**
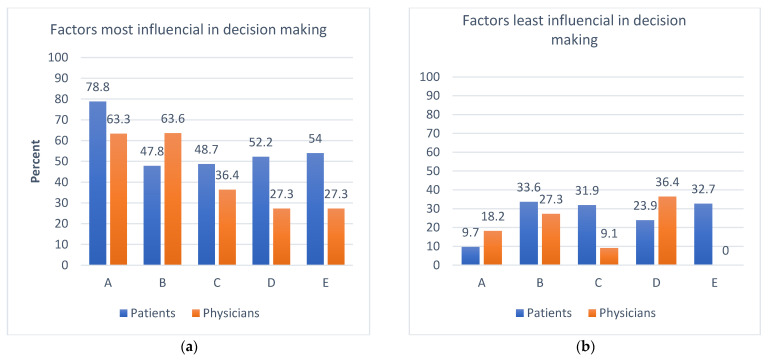
Patient vs physician interest in genomic testing. Responses to questions, respectively: Of the following listed factors, which would (**a**) most and (**b**) least influence you in a decision to pursue genomics testing in the care of your cancer patients? A. Potential to guide treatment selection; B. Potential to predict disease outcome; C. Potential to inform side effects management; D. Potential to learn more about my cancer; E. Desire to contribute to scientific research.

Concerning the factors that influence decision making, the potential to guide treatment influenced both the patients’ and physicians’ decisions the most (78.8%, *n* = 89 and 63.3%, *n* = 7, respectively). In addition, physicians were also found to be interested in the ability of genomic testing to predict the disease outcome (63.6%, *n* = 7). However, the views of patients were different from their oncologists with respect to the goals and expectations of genomic testing. Specifically, patients were more willing to know about their cancer and side effects from treatments and contribute to research, while physicians were more interested in patient outcomes (Figure 3a). Patients also seemed to be the least curious to know about the side effects and disease outcomes when making decisions about testing (Figure 3b).

Furthermore, the possibility of a significant complication from the procedure was an essential factor influencing the decision making for both groups (concordance 56.25%). Patients’ and physicians’ views were discrepant regarding genomic testing, causing delays in treatment (concordance 26.7%). This was instead considered an essential factor by the patients to decline genomic testing (41.6, *n*= 47) in contrast to this consideration for only 18% (*n* = 2) of physicians (concordance 0.89%). Physicians were more concerned about the impact of genomic testing on the social aspects of the patients’ lives, including the discrimination of health, life, and disability insurance (42.0%, *n* = 5), in contrast to patients (18.0%, *n* = 20), who were more worried that the test was of no clinical value (40.0%, *n* = 45). Importantly, knowledge and expectations regarding the applications of genomic test results on actual diagnosis and prognosis were grossly discrepant between patients and their oncologists (concordance 26.1% and 36.0%, respectively, Table 2).

## 4. Discussion

The advent of genomic testing capable of identifying clinically significant somatic mutations has revolutionized the world of cancer diagnosis and therapeutics. It helps to characterize tumours at the molecular level, thus enabling medical oncologists to take a more personalized approach to clinical care and optimize patient selection for clinical research. Several new targeted therapies are now widely used and playing critical roles in improving the survival of patients with various cancers. The growing need and application of genomic testing underscore the importance of investigating how well cancer patients understand, value, and assimilate this information. 

Patient–physician interaction is an integral part of cancer care delivery, and previous studies have shown that physician communication behavior impacts health outcomes [32,33]. The complexity of precision medicine complicates the exchange of information in significant ways. We found several deficiencies in patients’ understanding of the goal and utilization of genomic testing offered to them. In our study, patients reported dissatisfaction at the end of the informed consent process and demanded more dedicated counselling concerning this topic (Figure 2C,D). In our study, there was no significant correlation found between the patient’s level of education and knowledge of genomic testing, indicating that a patient’s literacy level can sometimes be misleading for the physicians and healthcare teams regarding the amount of information provided to them (Supplementary Table S1A,B). While most of our study patients understood that cancer results from changes in the DNA, known as “mutations,” many patients did not understand the differences between somatic and inherited mutations. Our findings are broadly consistent with previous studies that have also demonstrated patient knowledge gaps concerning their understanding of cancer biology [21,30]. For instance, an analysis of breast cancer patients conducted by Lillie et al. showed that after reading written information about genomic testing, patients with limited health literacy experienced poor recall and were less likely to actively participate in decision making, leading to poor outcomes [34,35]. Therefore, physicians’ lack of awareness about their patients could easily lead to misinterpretation of test results by the patients, as well as create incorrect perceptions about its applications.

Therefore, the current informed consent process warrants close examination, since there appears to be a discrepancy between what is conveyed and what is understood by patients. There should be frank, patient-centered, and empathetic discussions aligned with the patient’s wishes and realistic goals [36,37,38]. Such a panel should be modified and simplified to help patients appropriately interpret the test results in their contexts and guide them in making decisions about their care. Emphasis should be placed on using simple language, regardless of their education level, to help patients understand their disease, the role of genomic testing, and its implications. Knowledge checks should supplement this during and at the end of the conversation. Appropriate communication tools, such as patient handouts, videos, or online teaching modules, should be used to deliver complex information to help patients better understand the applications of genomic tests [38]. The use of technology to develop practical communication tools, implement evidence-based decision aids for patients, and create online patient portals to monitor the uptake of such interventions should be promoted [31,38,39].

It is also essential to know if the person obtaining the consent has enough knowledge and the required communication skills to share complex information and tailor it to an individual patient’s needs and circumstances [39]. Physicians could also help by ensuring that they feel comfortable having these difficult conversations by educating themselves about the current genomic interventions and their applications. A multilevel interventional approach to patient communication was implemented in the VOICE study [40], showing the tremendous success of multidisciplinary approaches and the value of training physicians in communication skills. We need to invest in interventions focused on patient-centered communication skills training for physicians and other healthcare teams (e.g., genetic counsellors) [32,39]. There is also a role for educating the allied healthcare professionals, including the nurses, with whom patients frequently get in touch to clarify some of the information exchanged during the consultation with their oncologists [39]. Patients with a family history of hereditary cancers should be referred to genetic counsellors, and appropriate guidance should be provided in case of reluctance by involving multidisciplinary teams [38]. In addition, such interventions can be beneficial for physicians, who often lack a background in genetic counselling and testing and are often tasked with interpreting results without assistance [32].

Furthermore, one must remember that patients with advanced cancer are often desperate about their prognosis and the diagnostic and treatment choices offered to them [32,38,40]. Consequently, patients often have unrealistic expectations from test results regarding prognosis and cure. In our study, patients were found to overestimate the offered tests’ benefits. On the other hand, the vast majority were willing to learn about cancer risk in their family members (Figure 2B). They believed that genomic information about their cancer could benefit their family members, as well. Still, they did not realize they were also at risk of receiving unexpected results about unknown but clinically relevant oncological mutations. Unfortunately, many of these mutations are rare and do not yet have therapies. Hence, it is crucial to assess each patient’s expectations of personal benefit and benefit to their family members and align them to the treatment goals.

## 5. Limitations

Our study has highlighted important differences in perceptions of genomic testing among patients and physicians. However, the results of this study should be interpreted in light of certain limitations. First, given the small sample size of patients, physicians, and tumour sites included in the study, participants in the study may not be representative of the total pool of patients offered genomic testing and physicians who use genomic testing in their clinical decision making. This is also true of the tumour sites and stages, as some cancer types have more applications of genomic testing than others. Additional details of the stage of cancer, types of the genomic test, and the number of patients who received targeted therapies following the use of genomic testing could also have impacted the study results. Second, we selected random patients from different cancer clinics; the patients who agreed to participate in the survey may be more knowledgeable than others. Third, the questions in the survey might not have been sensitive to individual cancer types. Finally, we did not explore individual patients’ motivations to undergo genomic testing.

## 6. Conclusions

Patients and cancer physicians are aware of the advances in precision oncology and are willing to participate in genomic testing and research. However, they did not consistently agree about the roles and applications of these tests, which may result in misplaced expectations. The results of our study point to the need for improved physician–patient interactions, backed by appropriate patient education regarding tumour genomic testing. There is a need to evaluate and improve existing communication strategies. Emphasis should be placed on developing interventions to educate patients and healthcare professionals regarding applications of genomic testing so that the decisions to undergo testing are based on appropriate knowledge and understanding of risks and benefits. Continual exploration of expectations and goals is needed from both sides of the patient–physician relationship to improve the quality of clinical decision making. 

## Figures and Tables

**Table 1:** Baseline characteristics of patients.

Demographic Characteristics	N (%)
**Gender**	
Male	61 (53.9%)
Female	52 (46%)
**Tumour sites**	
Breast	48 (44%)
Colon	30 (26.5%)
Pancreas	5 (4.4%)
Renal	4 (3.5%)
Melanoma	11 (9.7%)
Lung	10 (8.8%)
Prostate	5 (4.4%)
**Marital status**	
Single	16 (14.2%)
Married	74 (65.5%)
Common-law/live-in partner	8 (7.1%)
Separated/divorced	6 (5.3%)
Widowed	7 (6.2%)
Other	1 (0.9%)
Prefer not to say	1 (0.9%)
**Highest level of education**	
Less than high school	1 (0.9%)
High school certificate or equivalent	26 (23%)
Community college, technical college, or CEGEP	41 (36.3%)
University—undergraduate degree	22 (19.5%)
University—graduate or professional degree	20 (17.7%)
Prefer not to say	3 (2.6%)
**Work experience/educational experience in healthcare**	
Yes	21 (18.6%)
No	92 (81.4%)
**A family member works in healthcare**	
Yes	39 (34.5%)
No	74 (65.4%)
**A family member diagnosed with cancer**	
Yes	62 (54.9%)
No	51 (45.1%)
**Personal annual income**	
Less than CAD 50,000	33 (29.2%)
CAD 50,000–99,999	34 (31.1%)
CAD 100,000–149,999	10 (8.8%)
CAD 150,000 or greater	6 (5.3%)
I prefer not to say	30 (26.5%)

**Table 2:** Concordance rates (%) for patients’ and physicians’ responses to questions about their interests, attitudes, and expectations toward genomic testing.

Questions about Patients’ and Physicians’ Interests, Attitudes, and Expectations about Genomics Testing in Cancer Care	Concordance (%)
Would most of your patients be willing to undergo a minor procedure—a needle biopsy under a local anesthetic, if required—to obtain a tissue sample for genomics testing?	68.75
Would most of your patients be willing to undergo a more significant procedure—a surgical biopsy under a general anesthetic—to obtain a tissue sample for genomics testing?	33.04
How much do you agree or disagree with the following statement about genomics testing in your cancer patients:	
Genomics testing based on today’s technology would significantly improve the DIAGNOSIS of a patient’s cancer.”	26.13
b. Genomics testing based on today’s technology would significantly improve the TREATMENT of a patient’s cancer.”	29.73
c. Genomics testing based on today’s technology would significantly improve a patient’s SURVIVAL from cancer.”	36.04
d. Genomics testing based on today’s technology would significantly improve a patient’s SIDE EFFECTS management from cancer treatment.”	25.23
Of the following listed factors, which would most influence you to pursue genomics testing in the care of your cancer patients?	
Potential to guide treatment selection	65.18
b. Potential to predict disease outcome	38.39
c. Likely to inform side effects management	23.21
d. Potential to learn more about the patient’s cancer	12.5
e. Desire to contribute to scientific research	None
Of the following listed factors, which would least influence you to pursue genomics testing in the care of your cancer patients?	
Potential to guide treatment selection	None
b. Potential to predict disease outcome	3.57
c. Likely to inform side effects management	5.36
d. Potential to learn more about the patient’s cancer	8.04
e. Desire to contribute to scientific research	13.39
Of the following listed factors, which would most influence you in a decision not to pursue genomics testing in the care of your cancer patients?	
Potential for a serious complication from a tissue biopsy	56.25
b. Potential of test results to be of no clinical value	19.64
c. Potential of test results to lead to discrimination of health, life, or disability insurance coverage	8.04
d. Concerns about the privacy and confidentiality of test results	None
e. Potential for a delay in treatment while awaiting test results	26.79
Of the following listed factors, which would least influence you in a decision not to pursue DNA testing in the care of your cancer patients?	
Potential for a serious complication from a tissue biopsy	6.25
b. Potential of test results to be of no clinical value	8.93
c. Potential of test results to lead to discrimination of health, life, or disability insurance coverage	5.36
d. Concerns about the privacy and confidentiality of test results	24.11
e. Potential for a delay in treatment while awaiting test results	0.89

## Data Availability

The data presented in this study are available on request from the corresponding author.

## Supplementary Materials

The following supporting information can be downloaded at: https://www.mdpi.com/article/10.3390/curroncol29120780/s1. Supplementary File S1: patient survey questionnaire; Supplementary File S2: physician survey questionnaire; Supplementary File S3: Table S1A: Comparison of patients’ literacy level (Q31 of patient survey, at or above high school level) and their knowledge of genomic testing (Q15a & b of patient survey) using the Pearson chi-square test; Table S1B: Comparison of patients’ annual income (Q35 of patient survey, A. <50,000 CAD B. >/= 50,000 CAD) and their knowledge of genomic testing (Q15a & b of patient survey) using the Pearson chi-square test.

## References

[1] McDermott U., Downing J.R., Stratton M.R. (2011). Genomics and the Continuum of Cancer Care. N. Engl. J. Med..

[2] Bombard Y., Bach P.B., Offit K. (2013). Translating Genomics in Cancer Care. J. Natl. Compr. Cancer Netw..

[3] Schwartzberg L., Kim E.S., Liu D., Schrag D. (2017). Precision Oncology: Who, How, What, When, and When Not?. Am. Soc. Clin. Oncol. Educ. Book.

[4] Seyhan A.A., Carini C. (2019). Are innovation and new technologies in precision medicine paving a new era in patient-centric care?. J. Transl. Med..

[5] Chevallier M., Borgeaud M., Addeo A., Friedlaender A. (2021). Oncogenic driver mutations in non-small cell lung cancer: Past, present and future. World J. Clin. Oncol..

[6] Ettinger D.S., Wood D.E., Aisner D.L., Akerley W., Bauman J.R., Bharat A., Bruno D.S., Chang J.Y., Chirieac L.R., D’Amico T.A. (2021). NCCN Guidelines Insights: Non–Small Cell Lung Cancer, Version 2.2021. J. Natl. Compr. Cancer Netw..

[7] Herbst R.S., Baas P., Kim D.-W., Felip E., Pérez-Gracia J.L., Han J.-Y., Molina J., Kim J.-H., Arvis C.D., Ahn M.-J. (2016). Pembrolizumab versus docetaxel for previously treated, PD-L1-positive, advanced non-small-cell lung cancer (KEYNOTE-010): A randomised controlled trial. Lancet.

[8] Kosaka T., Yatabe Y., Endoh H., Yoshida K., Hida T., Tsuboi M., Tada H., Kuwano H., Mitsudomi T. (2006). Analysis of Epidermal Growth Factor Receptor Gene Mutation in Patients with Non–Small Cell Lung Cancer and Acquired Resistance to Gefitinib. Clin. Cancer Res..

[9] Karapetis C.S., Khambata-Ford S., Jonker D.J., O’Callaghan C.J., Tu D., Tebbutt N.C., Simes R.J., Chalchal H., Shapiro J.D., Robitaille S. (2008). K-rasMutations and Benefit from Cetuximab in Advanced Colorectal Cancer. N. Engl. J. Med..

[10] Bokemeyer C., Bondarenko I., Hartmann J.T., De Braud F., Schuch G., Zubel A., Celik I., Schlichting M., Koralewski P. (2011). Efficacy according to biomarker status of cetuximab plus FOLFOX-4 as first-line treatment for metastatic colorectal cancer: The OPUS study. Ann. Oncol..

[11] Van Cutsem E., Köhne C.-H., Láng I., Folprecht G., Nowacki M.P., Cascinu S., Shchepotin I., Maurel J., Cunningham D., Tejpar S. (2011). Cetuximab Plus Irinotecan, Fluorouracil, and Leucovorin As First-Line Treatment for Metastatic Colorectal Cancer: Updated Analysis of Overall Survival According to Tumor KRAS and BRAF Mutation Status. J. Clin. Oncol..

[12] Ogunwobi O.O., Mahmood F., Akingboye A. (2020). Biomarkers in Colorectal Cancer: Current Research and Future Prospects. Int. J. Mol. Sci..

[13] Artomov M. (2019). Improving survival prediction for melanoma. eLife.

[14] Korngiebel D.M., Thummel K.E., Burke W. (2017). Implementing Precision Medicine: The Ethical Challenges. Trends Pharmacol. Sci..

[15] Lerman C. (1996). BRCA1 Testing in Families With Hereditary Breast-Ovarian Cancer. JAMA.

[16] Donovan K.A., Tucker D.C. (2000). Knowledge about genetic risk for breast cancer and perceptions of genetic testing in a sociodemographically diverse sample. J. Behav. Med..

[17] Cyrus-David M.S. (2010). Knowledge and Accuracy of Perceived Personal Risk in Underserved Women Who are at Increased Risk of Breast Cancer. J. Cancer Educ..

[18] Caruso A., Vigna C., Maggi G., Sega F.M., Cognetti F., Savarese A. (2008). The withdrawal from oncogenetic counselling and testing for hereditary and familial breast and ovarian cancer. A descriptive study of an Italian sample. J. Exp. Clin. Cancer Res..

[19] Bluman L.G., Rimer B., Berry D.A., Borstelmann N.A., Iglehart J.D., Regan K., Schildkraut J.M., Winer E.P. (1999). Attitudes, knowledge, and risk perceptions of women with breast and/or ovarian cancer considering testing for BRCA1 and BRCA2. J. Clin. Oncol. Off. J. Am. Soc. Clin. Oncol..

[20] Gray S.W., Hicks-Courant K., Lathan C.S., Garraway L., Park E.R., Weeks J.C. (2012). Attitudes of Patients With Cancer About Personalized Medicine and Somatic Genetic Testing. J. Oncol. Pract..

[21] Cheung W.Y., Neville B.A., Cameron D.B., Cook E.F., Earle C.C. (2009). Comparisons of Patient and Physician Expectations for Cancer Survivorship Care. J. Clin. Oncol..

[22] Cheung W.Y., Neville B.A., Earle C.C. (2010). Associations Among Cancer Survivorship Discussions, Patient and Physician Expectations, and Receipt of Follow-Up Care. J. Clin. Oncol..

[23] Ciardiello F., Adams R., Tabernero J., Seufferlein T., Taieb J., Moiseyenko V., Ma B., Lopez G., Vansteenkiste J.F., Esser R. (2016). Awareness, Understanding, and Adoption of Precision Medicine to Deliver Personalized Treatment for Patients With Cancer: A Multinational Survey Comparison of Physicians and Patients. Oncologist.

[24] Lee I.-H., Kang H.-Y., Suh H.S., Lee S., Oh E.S., Jeong H. (2018). Awareness and attitude of the public toward personalized medicine in Korea. PLoS ONE.

[25] Kichko K., Marschall P., Flessa S. (2016). Personalized Medicine in the U.S. and Germany: Awareness, Acceptance, Use and Preconditions for the Wide Implementation into the Medical Standard. J. Pers. Med..

[26] Marchiano E.J., Birkeland A.C., Swiecicki P.L., Spector-Bagdady K., Shuman A.G. (2018). Revisiting Expectations in an Era of Precision Oncology. Oncologist.

[27] Bedard P.L., Oza A.M., Tsao M.-S., Leighl N.B., Shepherd F.A., Chen E.X., Tannock I., Krzyzanowska M.K., Dhani N.C., Clarke B. (2013). Princess Margaret Cancer Centre (PMCC) Integrated Molecular Profiling in Advanced Cancers Trial (IMPACT) using genotyping and targeted next-generation sequencing (NGS). J. Clin. Oncol..

[28] Dancey J.E., Bedard P.L., Onetto N., Hudson T.J. (2012). The Genetic Basis for Cancer Treatment Decisions. Cell.

[29] Miller F.A., Hayeems R.Z., Bytautas J.P., Bedard P.L., Ernst S., Hirte H., Hotte S., Oza A., Razak A., Welch S. (2014). Testing personalized medicine: Patient and physician expectations of next-generation genomic sequencing in late-stage cancer care. Eur. J. Hum. Genet..

[30] Blanchette P.S., Spreafico A., Miller F.A., Chan K., Bytautas J., Kang S., Bedard P.L., Eisen A., Potanina L., Holland J. (2014). Genomic testing in cancer: Patient knowledge, attitudes, and expectations. Cancer.

[31] Prince A.E.R., Berkman B.E. (2018). Reconceptualizing harms and benefits in the genomic age. Per. Med..

[32] Mcfarland D.C., Blackler E., Banerjee S., Holland J. (2017). Communicating About Precision Oncology. JCO Precis. Oncol..

[33] Arora N.K. (2003). Interacting with cancer patients: The significance of physicians’ communication behavior. Soc. Sci. Med..

[34] Pellegrini I., Rapti M., Extra J.M., Petri-Cal A., Apostolidis T., Ferrero J.-M., Bachelot T., Viens P., JuliaŽ Reynier C., Bertucci F. (2012). Tailored chemotherapy based on tumour gene expression analysis: Breast cancer patients’ misinterpretations and positive attitudes. Eur. J. Cancer Care.

[35] Lillie S.E., Brewer N.T., O’Neill S.C., Morrill E.F., Dees E.C., Carey L.A., Rimer B. (2007). Retention and Use of Breast Cancer Recurrence Risk Information from Genomic Tests: The Role of Health Literacy. Cancer Epidemiol. Biomark. Prev..

[36] McClement S.E., Chochinov H.M. (2008). Hope in advanced cancer patients. Eur. J. Cancer.

[37] Sulmasy D.P., Astrow A.B., He M.K., Seils D.M., Meropol N.J., Micco E., Weinfurt K.P. (2010). The culture of faith and hope. Cancer.

[38] Weeks J.C., Catalano P.J., Cronin A., Finkelman M.D., Mack J.W., Keating N.L., Schrag D. (2012). Patients’ Expectations about Effects of Chemotherapy for Advanced Cancer. N. Engl. J. Med..

[39] Kalia S.S., Adelman K., Bale S.J., Chung W.K., Eng C., Evans J.P., Herman G.E., Hufnagel S.B., Klein T.E., Korf B.R. (2017). Recommendations for reporting secondary findings in clinical exome and genome sequencing, 2016 update (ACMG SF 2.0): A policy statement of American College of Genetics and Genomics. Genet. Med..

[40] Hoerger M., Epstein R.M., Winters P.C., Fiscella K., Duberstein P.R., Gramling R., Butow P.N., Mohile S.G., Kaesberg P.R., Tang W. (2013). Values and options in cancer care (VOICE): Study design and rationale for patient-centred communication and decision-making intervention for physicians, patients with advanced cancer, and their caregivers. BMC Cancer.

