# Supplementary material for: A Comparison of Patients’ and Physicians’ Knowledge and Expectations Regarding Precision Oncology Tests
Source: Curr Oncol. 2022 Dec 16;29(12):9916–27. doi: 10.3390/curroncol29120780 (PMC9776922; doi:10.3390/curroncol29120780)
Supplement: Supplementary file 1 [file curroncol-29-00780-s001.zip › curroncol-1921596-supplementary files/curroncol-1921596-supplementary file S1.pdf]

UNIVERSITY OF CALGARY

SURVEY QUESTIONNAIRE

"ATTITUDES, EXPECTATIONS, AND KNOWLEDGE ABOUT PECISION ONCOLOGY AMONG PATIENTS AND PHYSICIANS"

This survey asks questions about cancer diagnosis and treatment using Precision Medicine, meaning use of genomics testing. Genomics testing can describe the genes within a patient's cancer cells, and results are currently being studied to help guide cancer therapy.
Genomics is the branch of research that focuses on DNA (deoxyribonucleic acid), which controls the appearance, behaviour, and function of cells. Genes can occasionally undergo changes that lead to cancer. New cancer therapies are being developed to fight against these genomics changes and using this information helps to improve treatment outcomes. We want to know how well our patients understand this information and what are their expectations from the newer therapies offered to them. Identifying the areas of agreement and disagreement between patients and providers can assist the development of strategies to improve education and counseling in future.

Section A: What is your general knowledge of cancer and it's testing?

The following questions pertain to your general understanding of cancer. Your responses can help us to better communicate and support future cancer patients.

1. Cancer represents uncontrolled cell growth.

O A. True
O B. False
O C. Don't know

2. All cancers are associated with poor outcomes.

O A. True
O B. False
O C. Don't know

3. Cancer results from a combination of inherited, environmental, and lifestyle factors.

O A. True
O B. False
O C. Don't know

4. Hereditary forms of cancer (inherited within families) are common

O A. True
O B. False
O C. Don't know

The following questions pertain to your understanding of how biological changes in your cells may cause cancer.

5. Genes are made from pieces of DNA (deoxyribonucleic acid).

O A. True
O B. False
O C. Don't know

6. A "mutation" refers to a change in DNA.

O A. True
O B. False
O C. Don't know

7. Any genomics change within a cell will give rise to cancer.

O A. True
O B. False
O C. Don't know

8. Cancers are usually caused by a change to a single gene.

O A. True
O B. False
O C. Don't know

The following questions pertain to your understanding of why genomics testing (mapping the mutations of your cancer) may be useful for cancer treatments.

9. Genomics testing can be used to identify genes that potentially play an important role in cancer

O A. True
O B. False
O C. Don't know

10. All cancers arising from the same organ have the same genomics characteristics.

O A. True
O B. False
O C. Don't know

11. Genomics testing can be used to help predict a patient's response to a specific cancer drug.

O A. True
O B. False
O C. Don't know

12. Genomics testing can be used to help predict how long a patient might live with cancer

O A. True
O B. False
O C. Don't know

13. Genomics testing (for acquired and inherited mutations) can potentially be used for which of the following.

O A. To confirm the type of cancer diagnosis
O B. To predict the risk of cancer for an individual, and their children or relatives
O C. To select treatment and monitor a patient's response to therapy
O D. To predict a disease outcome
O E. All of the above

Section B: Attitudes and expectations about cancer testing

We would also like to understand your attitudes and expectations about genomics testing in cancer care. We are only interested in your opinion, so there are no wrong answers.

14. Genomics testing is performed directly on a sample of the patient's cancer cells.

a) Would you be willing to undergo a minor procedure - a needle biopsy under a local anaesthetic, if required, to obtain a tissue sample for genomics testing?

O A. Yes
O B. No
O C. Unsure

b) Would you be willing to undergo a more major procedure - a surgical biopsy under a general anaesthetic, if required, to obtain a tissue sample for genomics testing?

O A. Yes
O B. No
O C. Unsure

15. How much do you agree or disagree with the following statement about genomics testing in cancer care:

a) "Genomics testing based on today's technology would significantly improve the DIAGNOSIS of my cancer."

O A. Strongly disagree
O B. Disagree
O C. Neither agree nor disagree
O D. Agree
O E. Strongly Agree

b) "Genomics testing based on today's technology would significantly improve the TREATMENT of my cancer."

O A. Strongly disagree
O B. Disagree
O C. Neither agree nor disagree
O D. Agree
O E. Strongly Agree

c) "Genomics testing based on today's technology would significantly improve the SURVIVAL from my cancer."

O A. Strongly disagree
O B. Disagree
O C. Neither agree nor disagree
O D. Agree
O E. Strongly Agree

d) "Genomics testing based on today's technology would significantly improve the SIDE EFFECTS of my cancer treatment."

O A. Strongly disagree
O B. Disagree
O C. Neither agree nor disagree
O D. Agree
O E. Strongly Agree

UNIVERSITY OF CALGARY

The following questions pertain to your interest in genomics testing for cancer care. We are only interested in your opinion, so there are no wrong answers.

16. Of the following listed factors, which would **most** influence you in a decision to pursue genomics testing in the care of your cancer? Please select up to 3 choices.

O A. Potential to guide treatment selection
O B. Potential to predict disease outcome
O C. Potential to inform side effects management
O D. Potential to learn more about my cancer
O E. Desire to contribute to scientific research
O F. Other (Please specify below)

____

____

____

____

17. Of the following listed factors, which would **least** influence you in a decision to pursue genomics testing in the care of your cancer?

O A. Potential to guide treatment selection
O B. Potential to predict disease outcome
O C. Potential to inform side effects management
O D. Potential to learn more about my cancer
O E. Desire to contribute to scientific research
O F. Other (Please specify below)

____

____

____

____

*The following questions pertain to why you may **not** wish to undergo genomics testing. We are only interested in your opinion, so there are no wrong answers.*

18. Of the following listed factors, which would **most** influence you in a decision **not** to pursue genomics testing in the care of your cancer? Please select up to 3 choices.

O A. Potential for a serious complication from a tissue biopsy
O B. Potential of test results to be of no clinical value
O C. Potential of test results to lead to discrimination of health, life or disability insurance coverage
O D. Concerns about the privacy and confidentiality of test results
O E. Potential for a delay in treatment while awaiting test results
O F. Other (Please specify below)

19. Of the following listed factors, which would **least** influence you in a decision **not** to pursue DNA testing in the care of your cancer?

O A. Potential for a serious complication from a tissue biopsy
O B. Potential of test results to be of no clinical value
O C. Potential of test results to lead to discrimination of health, life or disability insurance coverage
O D. Concerns about the privacy and confidentiality of test results
O E. Potential for a delay in treatment while awaiting test results
O F. Other (Please specify below)

The primary goal of genomics testing in cancer care is to help guide cancer treatment. However, other results may also be generated through testing which could have important health or reproductive implications for you/and or your family

We are only interested in your opinion about this possibility, so there are no wrong answers.

20. I would **only** want to receive genomics test results that would influence my current or future cancer treatment decisions.

O A. Yes
O B. No
O C. Unsure

21. I would want to receive genomics test results about my family's inherited risk of developing cancer.

O A. Yes
O B. No
O C. Unsure

22. I would want to receive genomics test results that would impact my risk of developing diseases **other** than cancer.

O A. Yes
O B. No
O C. Unsure

We are also interested in your attitude about sharing your genomics test results and biological (e.g. tissue, blood, etc.) samples for future scientific research. "Biobanking" is a term used for ongoing storage of test results and biological samples. The biobank could be located in your cancer centre, elsewhere in Canada, or around the world. The biobank contains information about the tissue that you have donated, but it does NOT contain any personal information.

We are only interested in your opinion, so there are no wrong answers.

23. Would you consent to biobank your genomics test results and tissue sample for future scientific research?

O A. Yes ➡ Please continue to Question 24
O B. No ➡ Please skip ahead to Question 27
O C. Unsure ➡ Please continue to Question 24

24. With whom would you be willing to share your genomics test results for scientific research?

O A. Research groups affiliated with my doctor
O B. Research groups affiliated with my hospital
O C. Other research groups within Canada
O D. Other research groups outside Canada
O E. Drug development or biotechnology companies
O F. All of the above

25. I would share my genomics test results and biological samples for any future cancer research, even if the research is not specifically related to my disease.

O A. Yes
O B. No
O C. Unsure

26. I would share my genomics test results and biological samples **only** for specific research studies where I have given written consent.

O A. Yes
O B. No
O C. Unsure

We are also interested in your comfort level to make an informed decision to pursue genomics testing for cancer.

We are only interested in your opinion, so there are no wrong answers.

27. Do you think you have sufficient knowledge regarding the potential benefits and risks of genomics testing in cancer care to make an informed decision to pursue testing?

O A. Yes
O B. No
O C. Unsure

28. Do you feel that you would need more formal counseling prior to pursuing genomics testing in the care of your cancer?

O A. Yes
O B. No
O C. Unsure

UNIVERSITY OF CALGARY

Section C: Your demographics

We would like to know more about you and your household. Your responses can help us to better communicate and support future cancer patients.

29. What is your marital status?

O A. Single (never married, no live-in partner)
O B. Married
O C. Common-law/live-in partner
O D. Separated/Divorced
O E. Widowed
O F. Other (*please specify*): ____________________
O G. Prefer not to say

30. Current or Past Occupation (*Please fill in the blank*): ____________________

31. What is the highest level of education that you have completed?

O A. Less than high school
O B. High school certificate or equivalent
O C. Community college, technical college, or CEGEP
O D. University – undergraduate degree
O E. University – graduate or professional degree
O F. Other (*please specify*): ____________________
O G. Prefer not to say

32. Do you have work experience or educational experience in health care?

O A. Yes
O B. No

33. Do you have a close family member: a parent, sibling or child who works or used to work in health care?

O A. Yes
O B. No

34. Do you have a close family member: a parent, sibling or child who has been diagnosed with cancer?

O A. Yes
O B. No

35. What is your personal annual income (before taxes)?

O A. Less than $50,000
O B. 50,000 - $99,999
O C. 100,000 - $149,999
O D. $150,000 or greater
O E. Prefer not to say

Thank you for your time and effort to complete this questionnaire.
Your responses will be very helpful.

We welcome your feedback about the questionnaire. Please use the space below.

Comments:

____

____

____

____

____

____
