# Supplementary material for: A Comparison of Patients’ and Physicians’ Knowledge and Expectations Regarding Precision Oncology Tests
Source: Curr Oncol. 2022 Dec 16;29(12):9916–27. doi: 10.3390/curroncol29120780 (PMC9776922; doi:10.3390/curroncol29120780)
Supplement: Supplementary file 1 [file curroncol-29-00780-s001.zip › curroncol-1921596-supplementary files/curroncol-1921596-supplementary file S2.pdf]

UNIVERSITY OF CALGARY

PHYSICIAN SURVEY QUESTIONNAIRE

This brief survey asks 6 questions about your views regarding genomics testing your cancer patients. It will take 5 minutes.

Section A: Attitudes and expectations about cancer testing

We would also like to understand your attitudes and expectations about genomics testing in your cancer patients.

1. Genomics testing typically requires patients to undergo biopsies since testing is performed directly on a sample of the patient's cancer cells.

a) In your opinion, would most of your patients be willing to undergo a minor procedure - a needle biopsy under a local anaesthetic, if required, to obtain a tissue sample for genomics testing?

O A. Yes
O B. No
O C. Unsure

b) In your opinion, would most of your patients be willing to undergo a more major procedure - a surgical biopsy under a general anaesthetic, if required, to obtain a tissue sample for genomics testing?

O A. Yes
O B. No
O C. Unsure

2. How much do you agree or disagree with the following statement about genomics testing in your cancer patients:

a) "Genomics testing based on today's technology would significantly improve the DIAGNOSIS of a patient's cancer."

O A. Strongly disagree
O B. Disagree
O C. Neither agree nor disagree
O D. Agree
O E. Strongly Agree

b) “Genomics testing based on today’s technology would significantly improve the TREATMENT of a patient’s cancer.”

O A. Strongly disagree
O B. Disagree
O C. Neither agree nor disagree
O D. Agree
O E. Strongly Agree

c) “Genomics testing based on today’s technology would significantly improve a patient’s SURVIVAL from cancer.”

O A. Strongly disagree
O B. Disagree
O C. Neither agree nor disagree
O D. Agree
O E. Strongly Agree

d) “Genomics testing based on today’s technology would significantly improve a patient’s SIDE EFFECTS management from cancer treatment.”

O A. Strongly disagree
O B. Disagree
O C. Neither agree nor disagree
O D. Agree
O E. Strongly Agree

The following questions pertain to your interest in genomics testing for cancer patients.

3. Of the following listed factors, which would **most** influence you in a decision to pursue genomics testing in the care of your cancer patients? Please select up to 3 choices.

O A. Potential to guide treatment selection
O B. Potential to predict disease outcome
O C. Potential to inform side effects management
O D. Potential to learn more about the patient’s cancer
O E. Desire to contribute to scientific research
O F. Other (Please specify below)

____

____

4. Of the following listed factors, which would **least** influence you in a decision to pursue genomics testing in the care of your cancer patients?

O A. Potential to guide treatment selection
O B. Potential to predict disease outcome
O C. Potential to inform side effects management
O D. Potential to learn more about the patient's cancer
O E. Desire to contribute to scientific research
O F. Other (Please specify below)

____

____

____

*The following questions pertain to why you may **not** wish to order genomics testing for your cancer patients.*

5. Of the following listed factors, which would **most** influence you in a decision **not** to pursue genomics testing in the care of your cancer patients? Please select up to 3 choices.

O A. Potential for a serious complication from a tissue biopsy
O B. Potential of test results to be of no clinical value
O C. Potential of test results to lead to discrimination of health, life or disability insurance coverage
O D. Concerns about the privacy and confidentiality of test results
O E. Potential for a delay in treatment while awaiting test results
O F. Other (Please specify below)

____

____

____

____

6. Of the following listed factors, which would **least** influence you in a decision **not** to pursue DNA testing in the care of your cancer patients?

O A. Potential for a serious complication from a tissue biopsy
O B. Potential of test results to be of no clinical value
O C. Potential of test results to lead to discrimination of health, life or disability insurance coverage
O D. Concerns about the privacy and confidentiality of test results
O E. Potential for a delay in treatment while awaiting test results
O F. Other (Please specify below)

____

____

Thank you for your time and effort to complete this questionnaire.
Your responses will be very helpful.

We welcome your feedback about the questionnaire. Please use the space below.

Comments:

____

____

____

____

____

____
