# Supplementary material for: A Comparison of Patients’ and Physicians’ Knowledge and Expectations Regarding Precision Oncology Tests
Source: Curr Oncol. 2022 Dec 16;29(12):9916–27. doi: 10.3390/curroncol29120780 (PMC9776922; doi:10.3390/curroncol29120780)
Supplement: Supplementary file 1 [file curroncol-29-00780-s001.zip › curroncol-1921596-supplementary files/curroncol-1921596-supplementary file S3.docx]

Supplementary Table S1:

**Table S1A**: Comparison of patients' literacy level (Q31 of patient survey, at or above high school level) and their knowledge of genomic testing (Q15a & b of patient survey) using the Pearson chi-square test. A value of <0.05 was considered significant.

| **Survey Questionnaire** | **Patient Response** | **Q31 At high school level** | **Q31 Diploma/University/equivalent** | **Chi-Square** | **p-value** |
| --- | --- | --- | --- | --- | --- |
| Patient knowledge Q15 a) | Agree | 17 | 49 | 0.049 | 0.824 |
|  | Disagree | 11 | 35 |  |  |
| Patient knowledge Q15 b) | Agree | 19 | 52 | 0.321 | 0.571 |
|  | Disagree | 9 | 32 |  |  |

Patient survey responses: Q31. What is the highest level of education that you have completed? A High school level B Diploma/University or equivalent. Q15 a) "Genomics testing based on today's technology would significantly improve the DIAGNOSIS of my cancer." Agree or disagree Q15 b) "Genomics testing based on today's technology would significantly improve the TREATMENT of my cancer." Agree or disagree. (Please refer to the patient survey, appendix 2) (response categories reduced for questions for statistical analysis).

**Table S1B**: Comparison of patients' annual income (Q35 of patient survey, A. <50,000 CAD B. >/= 50,000 CAD) and their knowledge of genomic testing (Q15a & b of patient survey) using the Pearson chi-square test. A value of <0.05 was considered significant.

| **Survey Questionnaire** | **Patient Response** | **Q35 Annual income**  **<50,000 CAD** | **Q35 Annual income**  **>/= 50,000 CAD** | **Chi-Square** | **p-value** |
| --- | --- | --- | --- | --- | --- |
| Patient knowledge Q15 a) | Agree | 23 | 43 | 2.042 | 0.153 |
|  | Disagree | 10 | 35 |  |  |
| Patient knowledge Q15 b) | Agree | 25 | 46 | 2.834 | 0.092 |
|  | Disagree | 8 | 32 |  |  |

Patient survey responses: Q35) What is your personal annual income? A. Less than $50,000 B. 50,000 - $99,999. Q15 a) "Genomics testing based on today's technology would significantly improve the DIAGNOSIS of my cancer." Agree or disagree (reduced category for statistical analysis). Q15 b) "Genomics testing based on today's technology would significantly improve the TREATMENT of my cancer." Agree or disagree. (Please refer to the patient survey, appendix 2) (response categories reduced for questions for statistical analysis).
